# Exploring the Anti-quorum Sensing and Antibiofilm Efficacy of Phytol against *Serratia marcescens* Associated Acute Pyelonephritis Infection in Wistar Rats

**DOI:** 10.3389/fcimb.2017.00498

**Published:** 2017-12-05

**Authors:** Ramanathan Srinivasan, Ramar Mohankumar, Arunachalam Kannappan, Veeramani Karthick Raja, Govindaraju Archunan, Shunmugiah Karutha Pandian, Kandasamy Ruckmani, Arumugam Veera Ravi

**Affiliations:** ^1^Department of Biotechnology, Alagappa University, Karaikudi, India; ^2^Department of Pharmaceutical Technology, National Facility for Drug Development for Academia, Pharmaceutical and Allied Industries, Bharathidasan Institute of Technology, Anna University, Tiruchirappalli, India; ^3^Department of Animal Science, Centre for Pheromone Technology, Bharathidasan University, Tiruchirappalli, India

**Keywords:** acute pyelonephritis, antibiofilm, anti-inflammatory agents, anti-quorum sensing, phytol, *Serratia marcescens*, Wistar rat

## Abstract

Quorum Sensing (QS) mechanism, a bacterial density-dependent gene expression system, governs the *Serratia marcescens* pathogenesis through the production of virulence factors and biofilm formation. The present study demonstrates the anti-quorum sensing (anti-QS), antibiofilm potential and *in vivo* protective effect of phytol, a diterpene alcohol broadly utilized as food additive and in therapeutics fields. *In vitro* treatment of phytol (5 and 10 μg/ml) showed decreasing level of biofilm formation, lipase and hemolysin production in *S. marcescens* compared to their respective controls. More, microscopic analyses confirmed the antibiofilm potential of phytol. The biofilm related phenomenons such as swarming motility and exopolysccharide productions were also inhibited by phytol. Furthermore, the real-time analysis elucidated the molecular mechanism of phytol which showed downregulation of *fimA, fimC, flhC, flhD, bsmB, pigP*, and *shlA* gene expressions. On the other hand, the *in vivo* rescue effect of phytol was assessed against *S. marcescens* associated acute pyelonephritis in Wistar rat. Compared to the infected and vehicle controls, the phytol treated groups (100 and 200 mg/kg) showed decreased level of bacterial counts in kidney, bladder tissues and urine samples on the 5th post infection day. As well, the phytol treatment showed reduced level of virulence enzymes such as lipase and protease productions compared to the infected and vehicle controls. Further, the infected and vehicle controls showed increasing level of inflammatory markers such as malondialdehyde (MDA), nitric oxide (NO) and myeloperoxidase (MPO) productions. In contrast, the phytol treatment showed decreasing level of inflammatory markers. In histopathology, the uninfected animal showed normal kidney and bladder structure, wherein, the infected animals showed extensive infiltration of neutrophils in kidney and bladder tissues. In contrast, the phytol treatment showed normal kidney and bladder tissues. Additionally, the toxic effect of phytol (200 mg/kg) was assessed by single dose toxicity analysis. No changes were observed in hematological, biochemical profiles and histopathological analysis of vital organs in phytol treated animals compared to the untreated controls. Hence, this study suggested the potential use of phytol for its anti-QS, antibiofilm and anti-inflammatory properties against *S. marcescens* infections and their associated inflammation reactions.

## Introduction

Urinary tract infection (UTI) is one among the utmost commonly detected infections in clinical settings (Hvidberg et al., 2000). In divergence to men, women are more vulnerable to UTI (Derbie et al., 2017). Almost 1 in 3 women will have had UTI by the age of 24 years. Nearly half of all female population will experience with UTI throughout their lifetime. The expenses have extended for the treatment of UTI infection is $2.4 billion a year by means of 4.5–6.8 million cases in worldwide (Foxman and Brown, 2003). The way of UTI is well-known, which starts from urethral to bladder and then moving up the ureters into the kidneys. Cystitis, a predominant UTI, takes place in the bladder of the lower urinary tract whereas the pyelonephritis, a severe kidney infection, that targets the upper urinary tract. Pyelonephritis is a potentially life threatening infection that often leads to renal damaging (Katsiari et al., 2012). Pyelonephritis occurs subsequently a sequence of events carrying the bacteria from outside of the human body, up to the bladder and finally settles down into the kidneys. If the pyelonephritis is not treated, this can lead to severe renal abscesses and sepsis along with renal failure (Ramakrishnan and Scheid, 2005). Most pyelonephritis infections are occurred by bacterial pathogens ascent through the urethra and urinary bladder. The etiologic agents of pyelonephritis are *Escherichia coli, Proteus mirabilis, Pseudomonas aeruginosa*, and *Serratia marcescens* (Ohno et al., 2003; Mittal et al., 2009; Chen et al., 2012; Kufel et al., 2016).

*Serratia marcescens*, a Gram-negative bacterium, belongs to the family Enterobacteriaceae, frequently isolated from urinary and respiratory tracts and it can function as an opportunistic pathogen in immunocompromised patients (Kida et al., 2007). The infections caused by *S. marcescens* are hard to treat since it possesses inherent resistance to an extensive variety of antibiotics (Lee et al., 2005; Kim et al., 2013; Leclercq et al., 2013; Liou et al., 2014; González-Juarbe et al., 2015). Development of antibiotic resistance in *S. marcescens* demands the urgent need for the alternative treatment approaches. Host-pathogen interaction and the ability of pathogens to modify the host response is a crucial factor for establishing successful infections (Youn et al., 1992). This capability of a pathogen is typically attributed to their ability to secrete several of virulence factors and to alter host immune response (McMillen et al., 1996). The prominence of these responses has been exposed in several biological processes through an assortment of inflammatory mediators and cytokines, which include tissue inflammation, wound healing and immune defense (McMillen et al., 1996; Rumbaugh et al., 2004). Recently, several reports specified that the quorum sensing (QS) mediated virulence factors are important for successful establishment of bacterial infection in animal models (Kumar et al., 2009; Gupta et al., 2013b). QS is a vital global gene regulatory machinery in bacteria that allows discrete bacteria to coordinate their virulence behavior in a cell density depended manner, which depends on self-produced signaling molecules called autoinducers (Rumbaugh et al., 1999). *Serratia marcescens* has a well described QS system (SmaI/SmaR) which utilizes different homoserine lactones (HSLs) such as C4-HSL, C6-HSL, and C8-HSL as signal molecules and governs the secretion of extensive range of virulence factors such as prodigiosin, lipase, protease, chitinase, nuclease, siderophore production, hemolysin production and most importantly biofilm formation (Hines et al., 1988; Eberl et al., 1996; Horng et al., 2002; Rice et al., 2005).

Research targeting the bacterial QS system has paid a great deal of attention for the identification of effective anti-quorum sensing (anti-QS) and antibiofilm agents. These anti-QS agents aim the virulence factors production rather the growth of the bacterial pathogen, hence the emergence of selective pressure for the development of antibiotic resistance strain is nullified. Thus, it is foreseen that the inhibition of such QS mechanism would warrant as an effective approach to reduce the *S. marcescens* pathogenicity and infection (Labbate et al., 2007). Recently, numerous studies have been continuously reported the anti-QS and antibiofilm potential of several natural compounds from plant origin. Plant sources play a vital role in delivering the novel drugs candidates in medicinal field. Phytol, a diterpene alcohol compound majorly found in essential oils, extensively used as fragrant ingredient in shampoos, cosmetics, fragrances and other toiletries (Islam et al., 2015). As well, it is also used in the production of Vitamin K and E. In therapeutic field, phytol has shown antioxidant and antinociceptive activities as well as antiallergic, antimicrobial, antiradical, anti-cholinesterase, antiamyloidogenic, and anti-inflammatory properties along with adequate safety (Inoue et al., 2005; Lim et al., 2006; Ryu et al., 2011; Santos et al., 2013; Pejin et al., 2014; Lee et al., 2016; Sathya et al., 2017). Also, the phytol is a tremendous immuno stimulant, in respect of long term memory stimulation of both acquired and innate immunity. However, the report on anti-QS potential of phytol against bacterial pathogens is very much scarce (Pejin et al., 2015) and the protective effect of phytol on bacterial pathogens in animal model is nil. Based on these facts, this pioneering study primarily focused on assessing the *in vitro* anti-QS and antibiofilm potential of phytol against *S. marcescens* and the *in vivo* protective effect of phytol against *S. marcescens* associated acute pyelonephritis infection in rat model.

## Materials and methods

### Uropathogenic *Serratia marcescens* and its growth conditions

*Serratia marcescens* strain PS1, a clinical strain isolated from urine sample collected from a clinical diagnosis laboratory in Meenakshi General Hospital, Chennai by Nithya et al. (2010) and identified through 16S rRNA gene sequencing with the GenBank accession number of FJ584421. *Serratia marcescens* was cultured in Luria- Bertani (LB) medium (pH 7.0) for overnight at 28°C. For the experimental purposes, the *S. marcescens* strain was sub cultured in LB medium until it reached 0.4 OD at 600 nm (1 × 10^8^ CFU/ml).

### Compound preparation

For *in vitro* study, one milligram of phytol (97%, mixture of isomers, catalog no. 139912, Sigma-Aldrich, St. Louis, MO, USA) was dissolved in 1 ml of methanol as stock solution and stored at 4°C till further use. For *in vivo* study, 200 mg of phytol was dissolved in 5 ml of corn oil as stock solution and stored at room temperature till further use. The maximum amount of methanol (10 μl, 1%) and corn oil (750 μl) was used as the vehicle controls (negative controls) for *in vitro* and *in vivo* assays, respectively.

### Biofilm cells quantification by XTT reduction assay

The effect of phytol on the metabolically active cells involved in biofilm formation of *S. marcescens* was evaluated by modified XTT reduction assay (Sivaranjani et al., 2016). The XTT sodium salt was prepared in phosphate buffer saline (PBS) at a concentration of 0.2 mg/ml and menadione in acetone at 0.172 mg/ml concentration. For each experiment, the XTT/menadione reagent was freshly prepared in the ratio of 12.5:1. *Serratia marcescens* culture was inoculated in 24-well microtitre plate (MTP) containing 1 ml of respective growth medium in the absence and presence of phytol (5&10 μg/ml) and incubated at 28°C for 24 h. Following incubation, the planktonic cells were discarded from 24-well MTP. Then, the biofilm cells on MTP wells were washed and resuspended in 200 μl of 0.9% NaCl. 25 μl of XTT-menadione solution was added in 96-well MTP containing biofilm cell suspensions and incubated at 37°C for 3 h in dark. Finally, the absorbance of biofilm cell suspensions together with XTT-menadione solution was measured at 490 nm by Multilabel Reader (Molecular devices, SpectraMax M3, USA).

### Growth curve analysis

The effect of phytol on *S. marcescens* growth was assessed by growth curve analysis. One percent of *S. marcescens* culture was added in to 100 ml of LB broth supplemented with (5 and 10 μg/ml) and without of phytol and the flasks were incubated in constant shaking at 120 rpm for 18 h in 28°C. The cell density was read at 600 nm for every 1 upto 18 h using Multilabel Reader (Packiavathy et al., 2013).

### Microscopic investigation of *S. marcescens* biofilm formation

To evaluate the antibiofilm potential of phytol, the light and confocal laser scanning microscopic (CLSM) analyses were done by following the method of Srinivasan et al. (2017a). After the growth of *S. marcescens* biofilm with and without of phytol on 1 × 1 cm glass slides, the planktonic cells were removed by washing with distilled water. Then the glass slides were stained with 0.4% crystal violet and 0.2% acridine orange for light and confocal microscopes, respectively. After 2 min of incubation, the excess stain was removed by distilled water wash and biofilms on glass slides were imaged under light (Nikon Eclipse Ti 100, Tokyo, Japan) and CLSM (Model LSM 710, Carl Zeiss, Oberkochen, Germany) at a magnification of 400 × and 200×, respectively. The Z-Stack CLSM images were analyzed using COMSTAT software to obtain the average thickness, biofilm biomass and surface to volume ratio of the phytol treated and untreated *S. marcescens* biofilm (Heydorn et al., 2000).

### Effect of phytol on *S. marcescens* swarming motility

The inhibitory effect of phytol on *S. marcescens* swarming motility was assessed by the method of Packiavathy et al. (2013). Briefly, the 5 μl of *S. marcescens* culture was inoculated in the center of swarming agar plate (1% peptone, 0.5% NaCl, and 0.5% agar) with the absence and presence of phytol (5 and 10 μg/ml). Then, the swarming plates were incubated for 16 h at 28°C and observed for inhibition in swarming motility.

### EPS quantification

Extraction of EPS from phytol treated and untreated *S. marcescens* culture was carried out by phenol-sulfuric acid method as described by Hirs (1967) with slight modification. Briefly, the *S. marcescens* culture was grown with the absence and presence of phytol (5 and 10 μg/ml) for 18 h at 28°C in 24 well MTP. After incubation, the planktonic cells were washed-out by sterile distilled water. Then the biofilm cells were dissolved by 0.9% NaCl (1 ml) and equilibrated phenol (1 ml). Afterward, 5 volume of H_2_SO_4_ was added to mix and incubated in dark at room temperature for 1 h. Then the absorbance was taken at 490 nm. The percentage of EPS inhibition was calculated by using the following formula.

$$\begin{array}{l} \left(\left(\text{ControlOD}-\text{TreatedOD}\right)/\text{ControlOD}\right)\times 100. \end{array}$$

### Lipase quantification assay

The phytol treated (5 and 10 μg/ml) and untreated *S. marcescens* culture was centrifuged at 10,000 rpm for 10 min. Then 100 μl of phytol treated and untreated CFCS was added to 900 μl of buffered substrate mixture having 9 volumes of 0.1% gummi arabicum and 0.2% sodium deoxycholate in 50 mM Na_2_PO_4_ buffer (pH 8.0) and 1 volume of 0.3% *p*-nitrophenyl palmitate in isopropanol and incubated at room temperature for 1 h. After incubation, the reaction was dismissed by adding 1 ml of 1 M Na_2_CO_3_ following which the mixture was centrifuged at 10,000 rpm for 10 min. Then, the absorbance of the supernatant was measured by Multilabel Reader at 410 nm (Srinivasan et al., 2017b). The percentage of lipase inhibition was calculated by using the formula as mentioned above.

### Haemolysin quantification assay

Fresh sheep blood was washed twice with PBS (pH 7.4) and resuspended in the same to a final concentration of 2% (v/v). To the 500 μl of 2% washed sheep erythrocytes, an equal volume of bacterial CFCS (treated with and without phytol) were added together and incubated at 37°C for 2 h. Then, the tubes were centrifuged and the hemolytic activity was determined by measuring the total amount of hemoglobin released in the supernatant at OD 405 nm in Multilabel Reader. The percent lysis was achieved by incubating the erythrocytes with distilled water (positive control) and background lysis was determined by incubating the erythrocytes with PBS (negative control). The percentage of lysis was determined by using the following formula (Kannappan et al., 2017).

$$\begin{array}{l} {}[({\text{A}}_{405}\text{ of sample}-{\text{A}}_{405}\text{ of background})/({\text{A}}_{405}\text{ of total}\\ \;-{\text{A}}_{405}\text{ of background})]\times 100. \end{array}$$

### Quantitative real-time PCR (qPCR) analysis

Total RNA was extracted from phytol treated (10 μg/ml) and untreated *S. marcescens* by TRIzol method and isolated RNA was converted into cDNA using Invitrogen — superscript III kit. qPCR was done on an Applied Biosystems thermal cycler by Power SYBR Green PCR master mix in 7500 Sequence Detection System (Applied Biosystems Inc. Foster, CA, USA). PCR cycles comprised an initial denaturation at 95°C for 10 min followed by 40 cycles of denaturation at 95°C for 45 s; annealing at 57°C for 45 s; extension at 72°C for 50 s. The expression patterns of candidate virulence genes were normalized against *rplU* gene (housekeeping gene) expression and quantified by calculating 2-ΔCt. Details of the primer sequences of the candidate and housekeeping genes (*fimA, fimC, flhC, flhD, bsmB, rssB, rsmA, pigP, shlA*, and *rplU*) used in this study are given in Table 1 and their efficiency was confirmed through 1.5% agarose gel electrophoresis (Supplementary Figure 1) (Salini and Pandian, 2015).

**Table 1:** Nucleotide sequences of *S. marcescens* primers used in this study.

**Gene**	**Primer sequence (5′-3′)**	
	**Forward**	**Reverse**
*rplU*	GCTTGGAAAAGCTGGACATC	TACGGTGGTGTTTACGACGA
*fimA*	ACTACACCCTGCGTTTCGAC	GCGTTAGAGTTTGCCTGACC
*fimC*	AAGATCGCACCGTACAAACC	TTTGCACCGCATAGTTCAAG
*flhC*	AAGAAGCCAAGGACATTCAG	TTCCCAGGTCATAAACCAGT
*flhD*	TGTCGGGATGGGGAATATGG	CGATAGCTCTTGCAGTAAATGG
*bsmB*	CCGCCTGCAAGAAAGAACTT	AGAGATCGACGGTCAGTTCC
*rssB*	TAACGAACTGCTGATGCTGT	GATCTTGCGCCGTAAATTAT
*rsmA*	TTGGTGAAACCCTCATGATT	GCTTCGGAATCAGTAAGTCG
*pigP*	GAACATGTTGGCAATGAAAA	ATGTAACCCAGGAATTGCAC
*shlA*	GCGGCGATAACTATCAAAAT	ATTGCCAGGAGTAGAACCAG

### *In vivo* therapeutic potential of phytol on *S. marcescens* associated acute pyelonephritis

#### Animals

Female Wistar rat (*Rattus norvegicus*) weighing 100–150 g, 6–8 weeks old were used in this study. They were kept in Central Animal House, Bharathidasan University, Tiruchirappalli, India. Rats were housed in polypropylene cages and were fed with standard rat synthetic diet (Sai Durga feeds, Bangalore) and water *ad libitum*. Ethical clearance was approved by the Institutional Animal Ethics Committee of Bharathidasan University, Tiruchirappalli, India (Approval ID: BDU/IAEC/2016/NE/37/Dt. 17.03.2016). All the experimental protocols were followed as per the guidelines of the Committee for the purpose of Control and Supervision of Experiments on Animals (CPCSEA), Government of India.

#### Establishment of experimental acute pyelonephritis in rat

**Figure 1:**
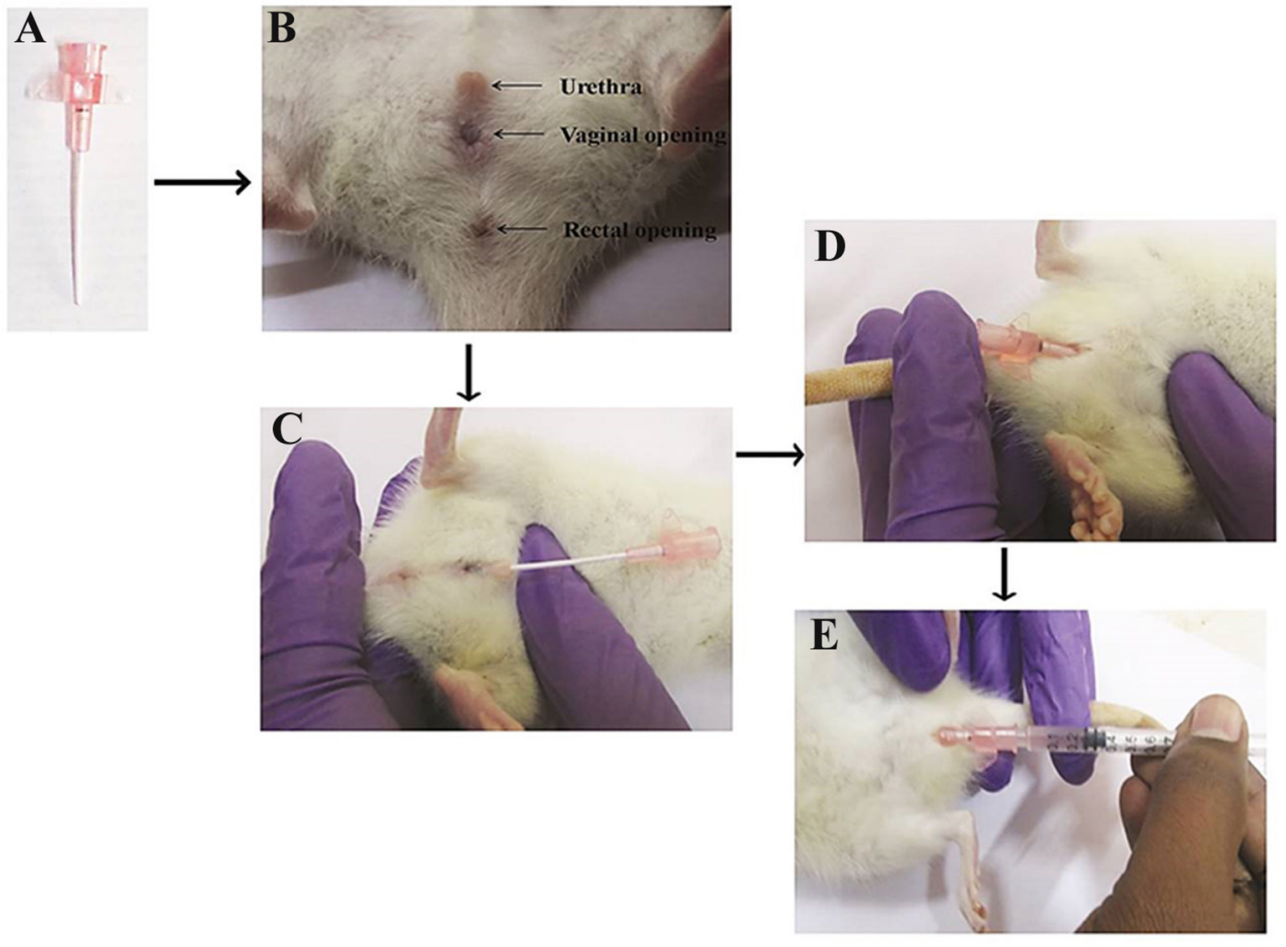
Urinary catheterization of angiocatheter into the bladder through the urethra. Twenty-gauge IV angiocatheter **(A)**, three visible urethral, vaginal and rectal opening **(B)**, insertion of angiocatheter into the urethral opening till it reaches the vaginal opening **(C)**, rotation of angiocatheter upward to the rat **(D)**, and injection of *S. marcescens* bacterial inoculum into the bladder **(E)**.

An experimental model of acute pyelonephritis infection was established in female Wistar rat as described by Brown (2011). Briefly, the rats were anesthetized with a ketamine-xylazine cocktail (90 mg ketamine + 9 mg xylazine) administered intraperitoneally at a dosage of 0.1 ml/100 g of body weight. Then, the rat was controlled in dorsal recumbency to facilitate way of the catheter. The rat body was hold in the nondominant hand with the tail positioned between the index and middle fingers and applies trivial pressure to the tail to spread. The exterior urethral orifice was identified. Then, the thumb has placed on the ventral stomach 1 cm forward to the urethral opening and gentle pressure was applied to pull the skin toward the head. This help to make the urethral opening further protruding as well to stretch the urethra to enable way of the catheter (Figure 1B). A small amount of lubricant was applied at the tip of the urethral orifice and the 20-gauge IV angiocatheter (Figure 1A) has inserted into the urethral opening in the direction of the tail till it reaches the vaginal opening (Figure 1C). After reaching the vaginal opening, the catheter was gently rotated upward to the rat (Figure 1D) and then 200 μl of *S. marcescens* bacterial inoculum (1 × 10^8^ CFU/ml) was gently injected into the bladder (Figure 1E) to avoid leak and reflux, kept in room for 10 min and then withdrawn prudently.

For experimental purpose animals were divided into four groups consisting of 5 animals each: (i) Group I — Infection control (Infection was given with *S. marcescens* cells to bladder through urethra); (ii) Group II — Vehicle control (Infection was given with *S. marcescens* cells and corn oil was given orally to infected animals after 24 h post infection (p.i.) until the 5th post-infection day (p.i.d.) for daily); (iii) Group III — Animals treated with phytol (Infected animals were treated daily with an oral dose of phytol (100 mg/kg body weight) after 24 h p.i. until the 5th p.i.d.); (iv) Group IV — Animals treated with phytol (Infected animals were treated daily with an oral dose of phytol (200 mg/kg body weight) after 24 h p.i. until the 5th p.i.d). After 5th p.i.d, the urine from each rat was collected in microfuge tubes by mild compression of the abdomen. Then the animals were sacrificed and organs were collected for further assays.

#### Bacteriological examination of urine, kidney, and bladder tissues

On the 5th p.i.d., animals from the all the groups were sacrificed. Kidney and bladder were removed aseptically, weighed and homogenized in 1 ml of phosphate buffered saline. Bacterial count was made afterward plating the appropriate dilutions of tissue homogenates and urine samples on Serratia differential agar (SD agar) plates (Himedia, India). Log bacterial counts were calculated per gram of tissue and per ml of urine as reported by Kumar et al. (2009). Further, the tissue homogenates were spun at 10,000 rpm for 10 min and filtered by 0.22 μm cellulose acetate membrane filter (Millipore, Bangalore, India). The obtained tissue homogenate filtrate was used for the estimation of protease and lipase production.

#### Estimation of protease production

Proteolytic activity was estimated by the method of Gupta et al. (2013a) with little modification. Briefly, the reaction mixture containing 200 μl of tissue homogenate diluted in 250 μl of buffered substrate [2% of azocasein (Sigma, USA) as substrate in 1 M Tris-HCl (pH-8.0)] was incubated at 37°C for 1 h. After subsequent incubation, the reaction mixture was added with 600 μl of 10% trichloro acetic acid to stop the reaction. The tubes were then spun at 10,000 rpm for 10 min and 600 μl of supernatant was added to 700 μl of 1 M NaOH. Absorbance was read at 440 nm in Multilabel reader and results were expressed in OD value.

#### Estimation of lipase production

Lipolytic activity of kidney and bladder tissue homogenized were determined using *p*-nitro phenyl palmitate (*p*-NPP) as the substrate. Two hundred microliter of tissue homogenate were added with 900 μl of reaction mix containing 1 volume of 0.3% *p*-NPP in propanol, 9 volumes of 0.1% gummi arabicum and 0.2% sodium deoxycholate in 50 mM Na_2_PO_4_ buffer (pH-8.0). The reaction mix was incubated for 1 h at room temperature in dark and then centrifuged at 10,000 rpm for 10 min. The reaction was dismissed by adding 1 ml of 1 M Na_2_CO_3_. Then, the absorbance was read at 410 nm and results were expressed in OD value (Srinivasan et al., 2017b).

#### Preparation of cell lysate

The kidney and bladder tissues from the experimental groups were homogenized using lysis buffer (10 mM Tris (pH-8.0), 20 mM EDTA and 0.25% Triton X-100). Then the supernatants were collected separately by centrifuging the tissue homogenate at 5,000 rpm for 30 min at 4°C and the protein quantification for the supernatant was done by Bradford method for all the samples. The cell lysate was kept at −80 °C until further analysis.

#### Malondialdehyde estimation

Induction of pathology was assessed on the base of malondialdehyde by the method of Ohkawa et al. (1979). Briefly, an equal volume of 10% ice-cold TCA was added to the cell lysate (protein concentration−100 μg) and centrifuged at 5,000 rpm for 15 min. MDA of five different concentrations (10–50 ng) were used as standard. To the supernatant and standard solution, same volume of 0.67% thiobarbituric acid in 50% glacial acetic acid was added and samples were incubated at 100°C for 20 min. After cooling, absorbance of the supernatant and standards were measured at 532 nm. The values were expressed as μM of TBARS/mg of protein determined by calibration curve prepared using different concentrations of MDA standards.

#### Quantification of myeloperoxidase (MPO) activity

Quantification of tissue neutrophils through the myeloperoxidase assay was done by the method of Kim et al. (2012) with slight modification. Briefly, the tissue homogenate was centrifuged at 8,000 rpm for 20 min at 4°C. The supernatant was discarded and 10 ml of ice-cold 50 mM potassium phosphate buffer (pH 6.0) comprising 0.5% hexadecyltrimethylammonium bromide and 10 mM EDTA was added to the pellet. It was then subjected to sonication and the solution was centrifuged at 10,000 rpm for 20 min. Then, 50 μl of supernatant was added with 50 μl of diluted H_2_O_2_ (4 μl of 30% H_2_O_2_ diluted in 96 μl of d H_2_O) and 200 μl of O-dianisidine mixture (16.7 mg of O-dianisidine, 90 ml of d H_2_O and 10 ml of potassium phosphate buffer). Three subsequent readings were taken at 450 nm at 30 s intervals. One unit of MPO defined as that degrading 1 μM of H_2_O_2_ per min at room temperature and myeloperoxidase activity was expressed as U/mg of tissue.

#### Estimation of nitrite content

Nitrite was estimated in the kidney and bladder tissues of experimental groups by the method of Rockett et al. (1994) with slight modification. Briefly, the cell lysate (100 μg of protein) in phosphate buffer (pH-7.4) was incubated with Griess reagent (Sigma Aldrich Chemicals Ltd., St Louis, MO, USA) for 30 min in dark at room temperature. The supernatant was collected and the optical density was measured at 540 nm along with the standard (5–20 μM sodium nitrite). The nitrite content was calculated with the help of sodium nitrite standard curve and the results were showed as μM of nitrite/mg of protein.

#### Histopathological analysis of kidney and bladder tissues

Kidney and bladder tissues were fixed in 10% buffered normal saline and dehydrated in gradient ethanol (30–100%). Paraffin wax blocks were prepared and thin sections were stained by hematoxylin and eosin. The pathological observations of all tissues were done through microscopy analysis by a pathologist.

### Pilot single-dose toxicity testing of phytol in Wistar rat

The rats were accommodated at a temperature of 25 ± 2°C in a 12 h light-dark cycle and acclimatized to laboratory conditions for 10 days without presenting any abnormality or pathological variations prior to experiments. Ten rats were arbitrarily divided into two groups; each containing five rats. The first group was the animal control which received the normal water, whereas the second group was orally administered with single dose of phytol (200 mg/kg body weight) for 14 days. The animals were observed for toxic signs for the first 2 h afterward dosing. Finally, the number of survivors was recorded after 24 h and animals were then maintained for additional 13 days with regular daily observations.

#### Hematological and biochemical analysis

On the day 15, all animals were anesthetized by urethane solution and blood samples were collected through retro-orbital puncture. Blood samples were collected into 2 tubes; heparinized and non-heparinized centrifuge tubes. The heparinized blood samples were used for a hematological study which includes hemoglobin concentration, white blood cell counts (WBC), red blood cell counts (RBC), and hematocrit. The serum detached from non-heparinized blood was used for a biochemical study which includes glucose, blood urea, creatinine, Alkaline phosphatase (ALP), Serum glutamic pyruvic transaminase (SGPT), Serum glutamic oxaloacetic transaminase (SGOT), triglycerides, Very low density lipoprotein (VLDL), Low density lipoprotein (LDL), High density lipoprotein (HDL), total bilirubin, direct bilirubin, indirect bilirubin, total cholesterol, total protein, albumin and globulin.

#### Histopathological analysis of vital organs

Immediately after collecting the blood samples, the vital organs such as kidney, liver, heart, lungs, and spleen were removed for histopathological analysis. Tissues from the animal control and the group treated with the phytol (200 mg/kg) were embedded in paraffin wax for sectioning. Further, the tissue sections were subjected to hematoxylin-eosin staining. The pathological observations of all tissues were performed through microscopic analysis by a pathologist.

#### Statistical analysis

All the *in vitro* experiments were conducted in triplicates and repeated thrice and the *in vivo* experiments were conducted in quintuplicates. The statistical analyses were done by SPSS statistics v17.0. Values were expressed as mean ± standard deviation. Student-*t* test was used to compare the control and treated samples.

## Results

### Quantification of biofilm cells by XTT reduction assay

**Figure 2:**
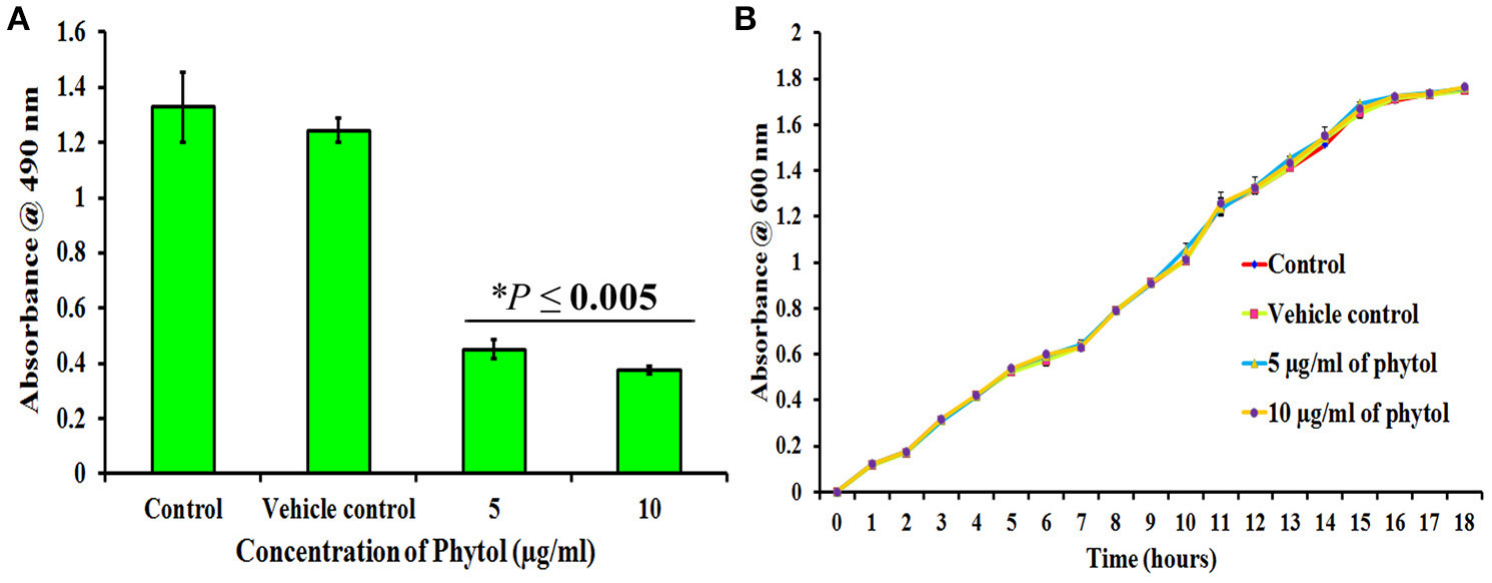
Effect of phytol on *S. marcescens* biofilm formation and growth. Phytol treatment (5 and 10 μg/ml) significantly inhibited the biofilm formation **(A)**, without affecting the growth of *S. marcescens* **(B)**. One percent of methanol was used as vehicle control. Error bar indicates standard deviations from the mean. Student-*t* test was used to compare the control and treated samples. ^*^Indicates significant at *p* ≤ 0.005.

The metabolically active cells involved in *S. marcescens* biofilm formation were quantified by XTT reduction assay. Results revealed that 5 and 10 μg/ml of phytol treatment showed lower level of optical density (OD 0.45 and 0.37, respectively) when compared to the untreated and vehicle controls (OD 1.32 and 1.24, respectively) (Figure 2A), which clearly indicates that phytol treatment reduces the number of metabolically active cells involved in biofilm formation.

### Effect of phytol on *S. marcescens* growth

To check the non-antibacterial activity of phytol, the bacterial growth curve assay was performed with *S. marcescens* in the absence and presence of phytol (5 and 10 μg/ml). Even after 18 h of incubation, no substantial differences were observed in the cell densities between untreated, vehicle controls and phytol treated samples (Figure 2B), which confirms that phytol did not have any antibacterial activity against *S. marcescens* at tested concentration.

### Light microscopic and CLSM analysis of *S. marcescens* biofilm formation

**Figure 3:**
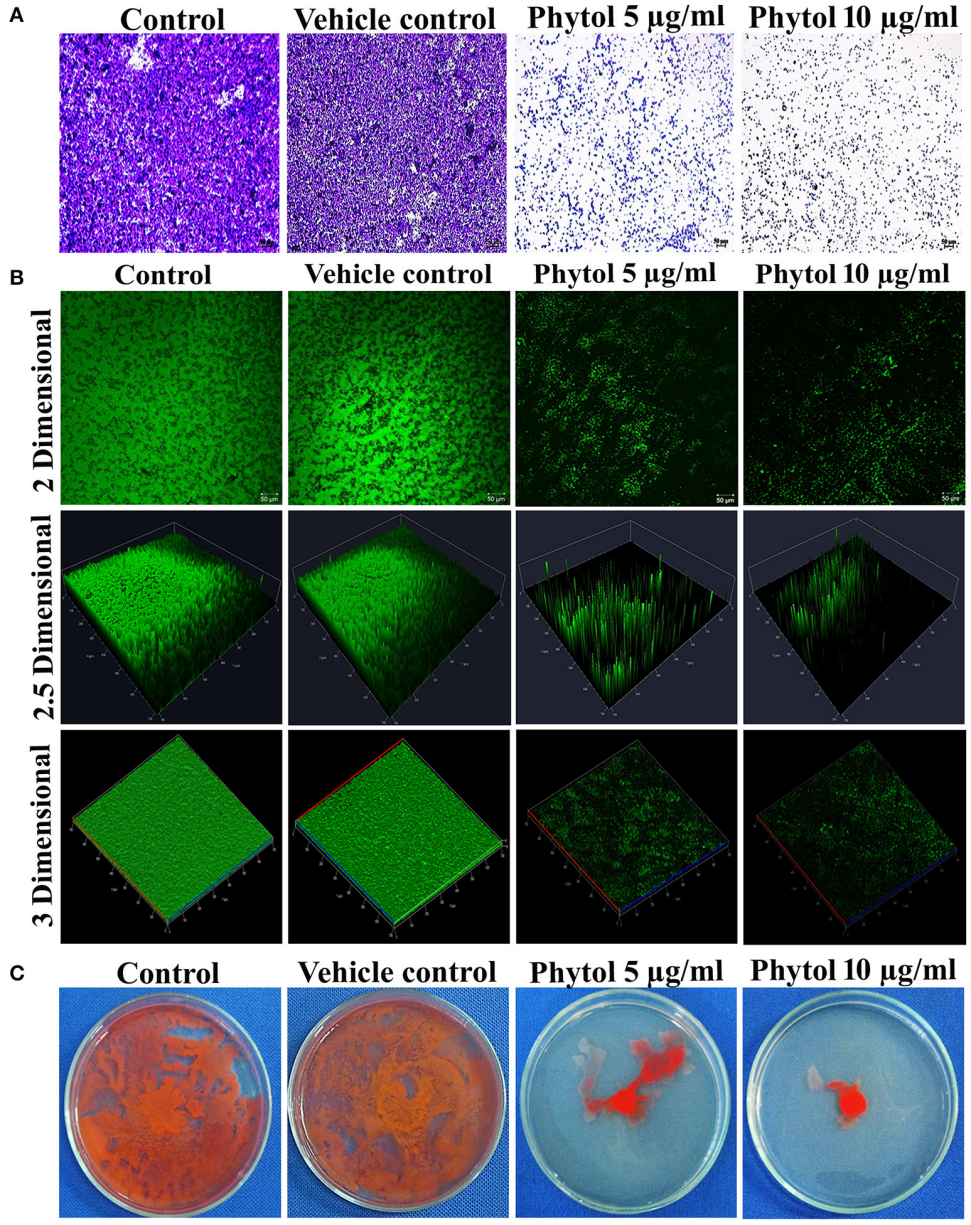
Microscopic analyses of *S. marcescens* biofilm formation. Light microscopic **(A)** and CLSM **(B)** images of phytol treatment slides (5 and 10 μg/ml) showed disintegration of *S. marcescens* biofilm formation compared to their untreated controls. Effect of phytol on *S. marcescens* swarming motility: The control plate exhibited extensive swarming motility on soft agar. In contrast, the phytol treatment (5 and 10 μg/ml) considerably inhibited the *S. marcescens* swarming motility **(C)**. One percent of methanol was used as a vehicle control.

Light microscopic observation of biofilm formation after treatment with phytol revealed their antibiofilm potential against *S. marcescens*. A thick coating of biofilm formation was observed in untreated and vehicle control samples, whereas a noticeable reduction of biofilm was observed in phytol treated samples (Figure 3A). In addition to this, the 2, 2.5, and 3 D CLSM images indicated the reduced in thickness and architecture of biofilms upon phytol treatment (Figure 3B). COMSTAT analysis was done to determine the 3D features like average thickness, biomass and surface volume ratio of *S. marcescens* biofilms with the absence and presence of phytol. The average thickness of the biofilm was reduced from 19.3 ± 0.29 to 12.1 ± 0.51 μm after treatment with phytol (10 μg/ml). Similarly, 5 and 10 μg/ml of phytol treatment showed reduced level of biofilm biomass (14.8 ± 0.38 and 12.0 ± 0.63 μm^3^/μm^2^, respectively) compare to the untreated and vehicle controls (20.0 ± 0.19 and 19.6 ± 0.4 μm^3^/μm^2^, respectively). Furthermore, 5 and 10 μg/ml of phytol treatment displayed increasing level of surface volume ratio (0.13 ± 0.08 and 0.20 ± 0.01, respectively) due to their biofilm disintegration property, wherein the surface volume ratio of untreated and vehicle control samples were 0.06 ± 0.01 and 0.07 ± 0, respectively (Table 2).

**Table 2:** COMSTAT analysis of phytol treated and untreated *S. marcescens* biofilm.

**Parameter**	**Control**	**Vehicle control**	**Phytol 5 μg/ml**	**Phytol 10 μg/ml**
Biomass (μm^3^/μm^2^)	20.08641 ± 0.19	19.68098 ± 0.4	14.85047 ± 0.38^*^	12.06844 ± 0.63^*^
Average thickness (μm)	19.3204 ± 0.29	19.12131 ± 0.59	14.78937 ± 0.14^*^	12.1517 ± 0.51^***^
Surface volume ratio (μm^2^/μm^3^)	0.06664 ± 0.01	0.07418 ± 0.00	0.131617 ± 0.08	0.203123 ± 0.01

*Phytol treatment (5 and 10 μg/ml) decreased the biofilm biomass, average thickness and increased the surface volume ratio of S. marcescens biofilm, compared to their untreated control. Data are expressed as mean ± SD. Student-t test was used to compare the control and treated samples*.

* *Indicates significant at p ≤ 0.005*.

*** *Indicates significant at p ≤ 0.0005*.

### Effect of phytol on *S. marcescens* swarming motility

Swarming motility is a QS mediated virulence attribute in *S. marcescens*. Obtained results clearly evident that the phytol (5 and 10 μg/ml) was able to reduce the *S. marcescens* swarming efficiency in a concentration dependent manner when compared to the untreated and vehicle controls (Figure 3C).

### Effect of phytol on EPS production

**Figure 4:**
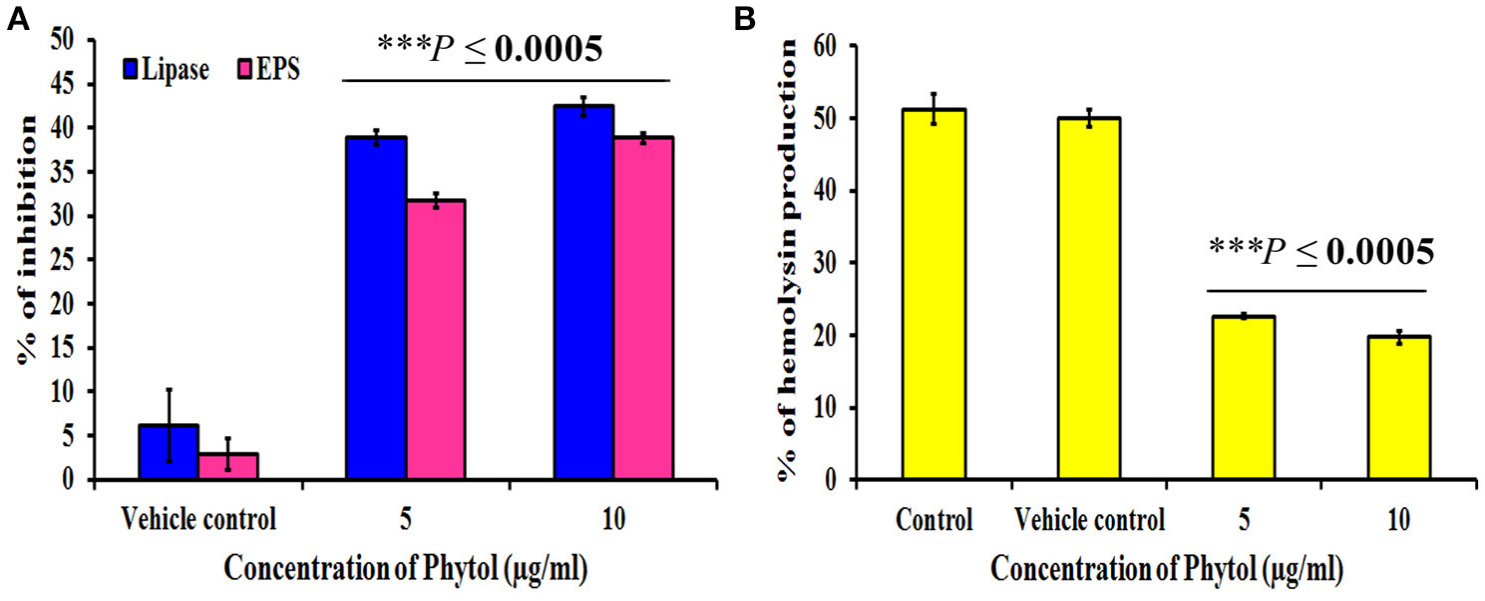
Effect of phytol on QS mediated virulence factors production in *S. marcescens*. Phytol treatment (5 and 10 μg/ml) significantly inhibited the lipase, EPS **(A)** and hemolysin **(B)** productions in *S. marcescens*. One percent of methanol was used as a vehicle control. Error bar indicates standard deviations from the mean. Student-*t* test was used to compare the control and treated samples. ^***^Indicates significant at *p* ≤ 0.0005.

Microbial cells cocooned themselves in self-secreted extra polymeric substances, which play a vital role in formation of biofilms. Phytol significantly (*P* ≤ 0.0005) inhibited the EPS production to the level of 32 and 39% at 5 and 10 μg/ml concentrations, respectively. Where, the vehicle control did not show any significant level of EPS inhibition (Figure 4A).

### Effect of phytol on lipase and hemolysin productions

*S. marcescens* is known to harbor important virulence factors including hemolysin production, which helps the bacteria in lysing human red blood cells and production of QS controlled extracellular virulence enzyme lipase. Therefore, the efficacy of phytol to inhibit the lipase and hemolytic virulence property of *S. marcescens* was assessed by lipolytic and hemolytic activities. The obtained results showed that phytol significantly (*P* ≤ 0.0005) inhibited the lipase and hemolysin productions to the level of 42 and 31% respectively, at 10 μg/ml concentration (Figures 4A,B).

### Expression of QS regulated genes in *S. marcescens* upon treatment with phytol

**Figure 5:**
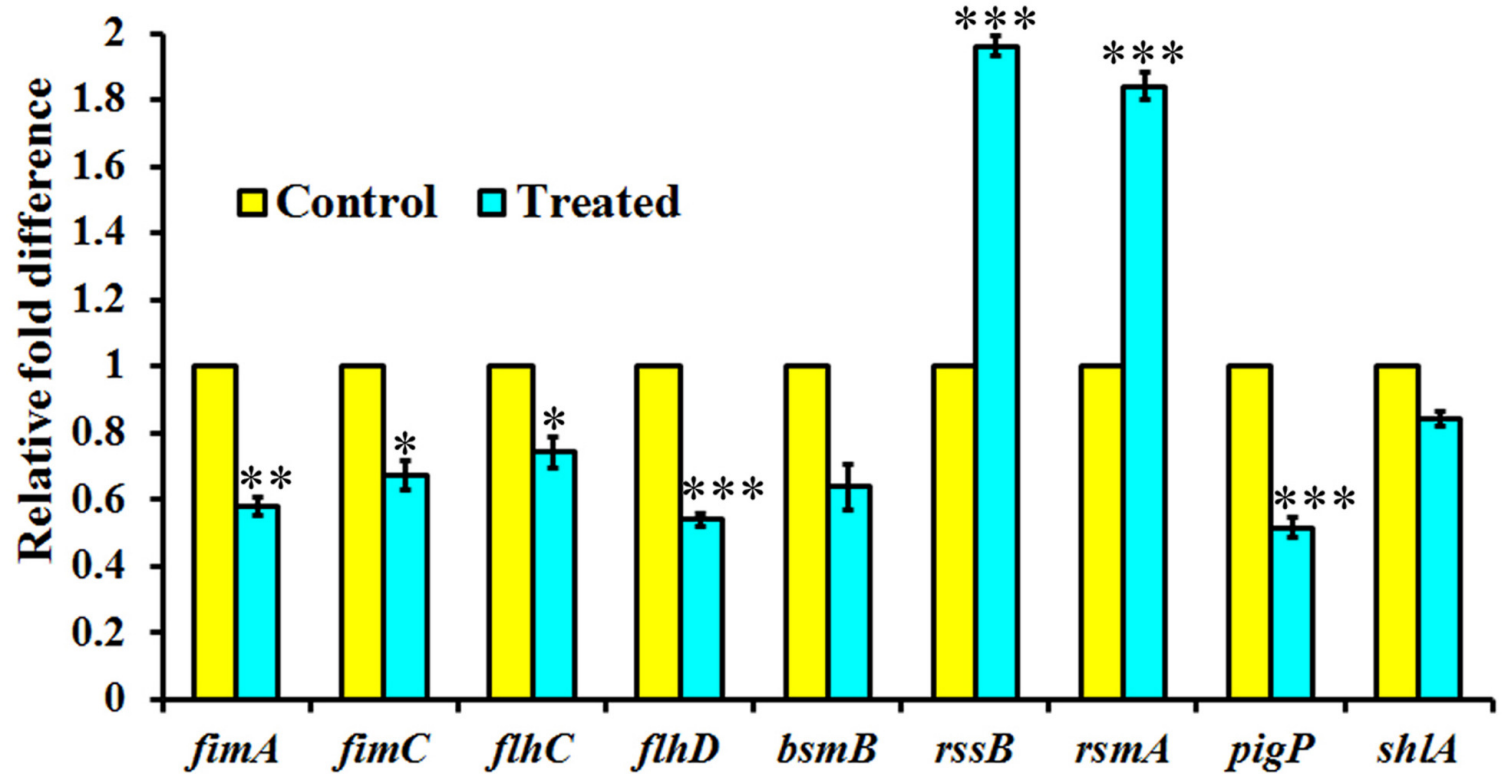
Effect of phytol treatment on the virulence gene expression of *S. marcescens*. Phytol treatment (10 μg/ml) modulated the expression of QS controlled genes involved in virulence factors production and biofilm formation in *S. marcescens*. Error bar indicates standard deviations from the mean. Student-*t* test was used to compare the control and treated samples. ^*^indicates significant at *p* ≤ 0.005, ^**^Indicates significant at *p* ≤ 0.001, and ^***^indicates significant at *p* ≤ 0.0005.

The expression level of QS-regulated genes was assessed in the *S. marcescens* in the presence of phytol (10 μg/ml) using real-time quantitative PCR. Phytol at tested concentration, downregulated the expression of *fimA, fimC, flhC, flhD, bsmB, pigP*, and *shlA* genes by 0.42, 0.32, 0.25, 0.46, 0.36, 0.48, and 0.15 fold, respectively in *S. marcescens* relative to the untreated controls. In divergence, phytol upregulated the *rssB* and *rsmA* gene expressions to the level of 0.96 and 0.84 fold respectively compared to the controls (Figure 5).

### *In vivo* protective effect of phytol on *S. marcescens* associated acute pyelonephritis

#### Morphological changes in kidney and bladder of infected and phytol treated animals

**Figure 6:**
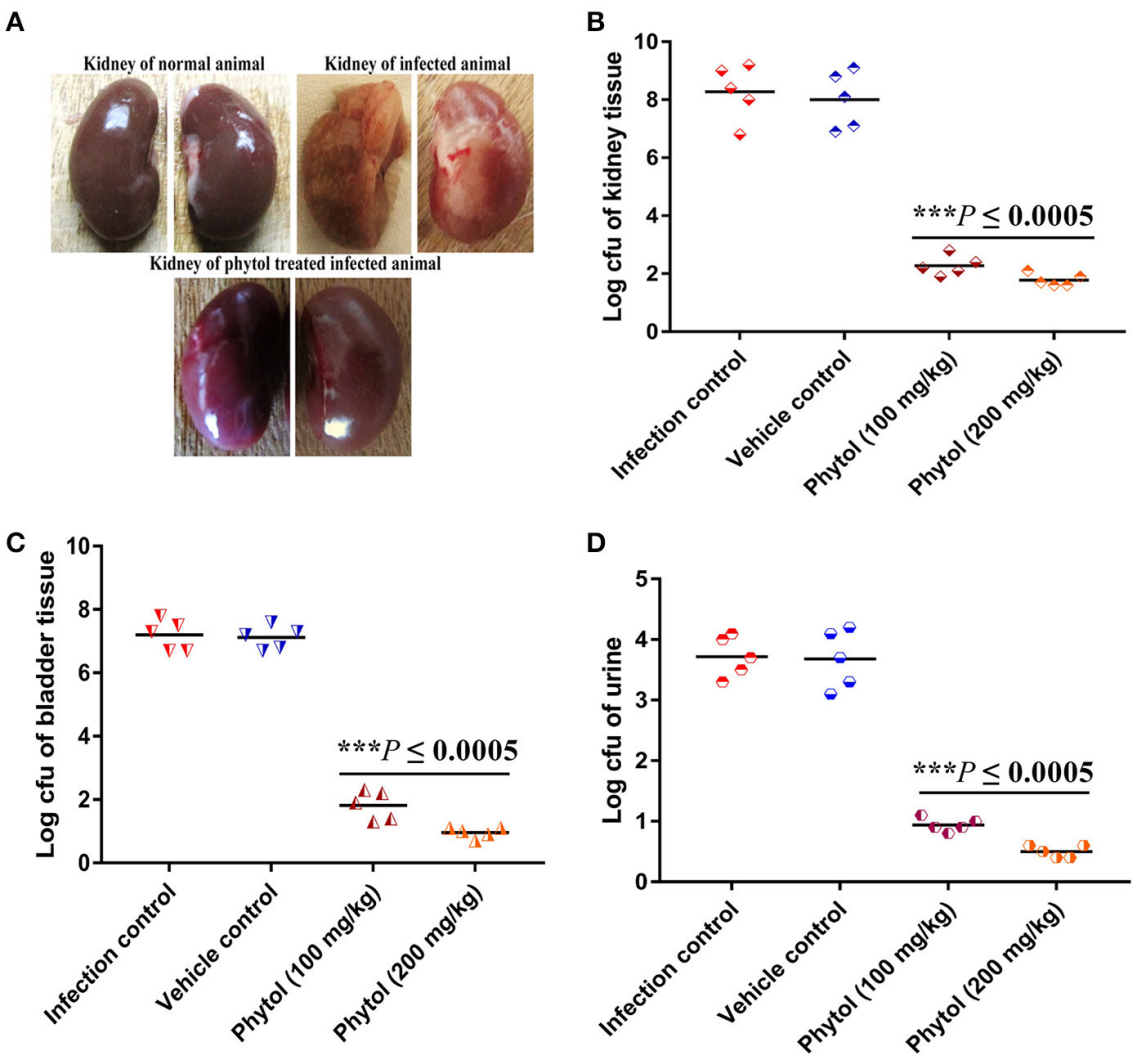
Morphological changes in rat kidney upon treatment with and without of phytol against *S. marcescens* associated acute pyelonephritis infection. The image of untreated infected animal showed damaged kidney along with pus formation and severe abscess. In contrast, the image of phytol treated infected animal showed healthy kidney like normal animal **(A)**. Quantitative bacterial load in kidney, bladder tissues, and urine samples: Compare to the infection controls, 100 and 200 mg/kg body weight of phytol treatment showed decreased level of bacterial load in kidney **(B)**, bladder **(C)** tissues, and urine **(D)** samples. Corn oil was used as the vehicle control. Data are expressed as mean ± SD. Student-*t* test was used to compare the control and treated samples. ^***^Indicates significant at *p* ≤ 0.0005.

Healthy kidney with smooth and normal bean shaped contours were observed in the normal uninfected rat, whereas, rat infected with *S. marcescens* by transurethral inoculation showed damaged kidney with severe abscess and pus formation. In contrast, the infected rat treated with phytol showed undamaged kidney which is similar like uninfected rat kidney (Figure 6A).

#### Assesment of bacterial burden in urine, kidney, and bladder tissues

The tissue homogenates of kidney, bladder and the urine samples were plated on SD agar plates for estimation of bacterial load. The 100 and 200 mg/kg body weight of phytol treatment showed a significant (*P* ≤ 0.0005) decline in kidney bacterial load by log 6 and 6.5, respectively on the 5th p.i.d compared to infected control group. On the other hand, a same level of bacterial load was observed in vehicle control group compared to the infected control (Figure 6B). A similar decreasing drift was observed with bladder and urine bacterial counts in phytol treated groups on the 5th p.i.d. The 100 and 200 mg/kg body weight of phytol treatment decreased the bladder bacterial count by log 5.3 and 6.2, respectively compared with the infected control group (Figure 6C). In urine sample, the 100 and 200 mg/kg body weight of phytol treatment decreased the bacterial count by log 2.7 and 3.2, respectively compared with the infected control group (Figure 6D).

#### Level of protease production in kidney and bladder tissues of phytol treated and untreated infected animals

**Figure 7:**
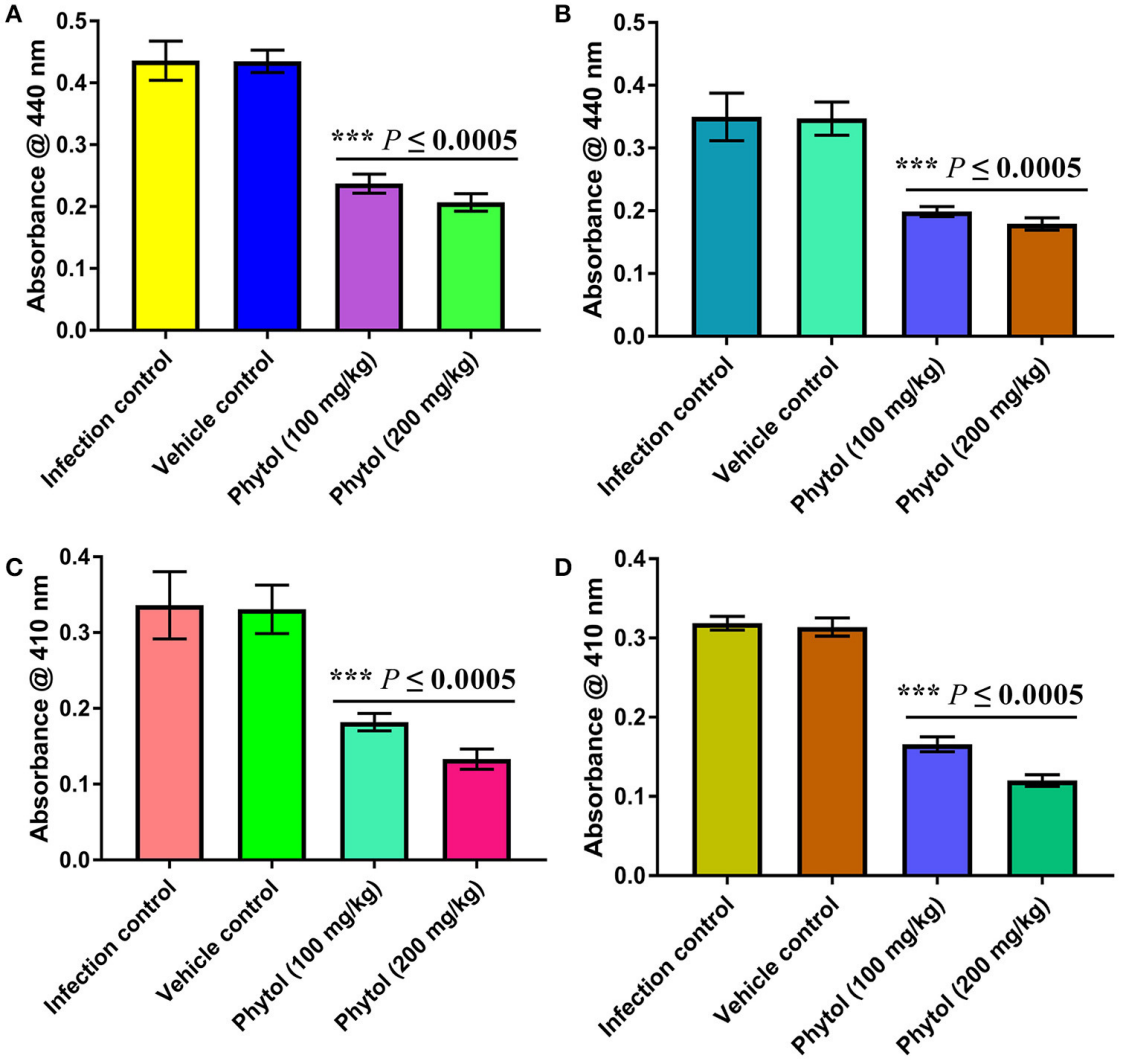
Inhibitory effect of phytol on protease and lipase productions in kidney and bladder tissues. Phytol treatment (100 and 200 mg/kg) significantly decreased the protease **(A,B)** and lipase **(C,D)** productions in kidney **(A,C)** and bladder **(B,D)** tissues of pyelonephritis induced rats, compared to their infection controls. Corn oil was used as the vehicle control. Error bar indicates standard deviations from the mean. Student-*t* test was used to compare the control and treated samples. ^***^Indicates significant at *p* ≤ 0.0005.

The protease is an extracellular virulence enzyme and its production is regulated by QS mechanism in *S. marcescens*. Therefore, the level of protease production in phytol treated and untreated kidney and bladder tissues were assessed. The results showed a decreased level of protease production in phytol treatment groups than the infected and vehicle controls (Figures 7A,B).

#### Level of lipase production in kidney and bladder tissues of phytol treated and untreated infected animals

Alike to protease, the production of an extracellular virulence lipase enzyme is controlled by QS mechanism. Therefore, the effect of phytol in lipase production of *S. marcescens* was assessed by lipolytic assay. The results revealed a decreased level of lipase production in phytol treatment groups compared to the infected and vehicle controls (Figures 7C,D).

#### Effect of phytol on MDA production

**Figure 8:**
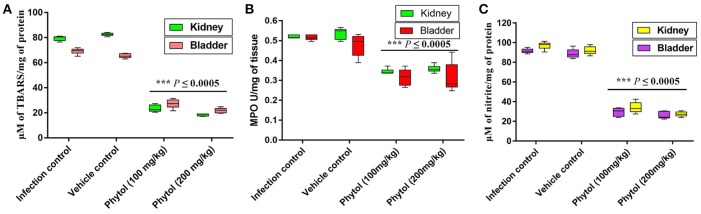
Levels of MDA, MPO, and NO productions in kidney and bladder tissues. Phytol treatment (100 and 200 mg/kg) significantly decreased the MDA **(A)**, MPO **(B)**, and NO **(C)** levels in kidney and bladder tissues of pyelonephritis induced rats, compared to their infection controls. Corn oil was used as the vehicle control. Error bar indicates standard deviations from the mean. Student-*t* test was used to compare the control and treated samples. ^***^Indicates significant at *p* ≤ 0.0005.

Free radicals mediated lipid peroxidation produces a large number of reactive aldehydes. MDA is one among the reactive aldehydes involved in pathophysiological modifications occurred during oxidative stress in tissues. Hence, the level of MDA production was estimated to assess the level of cellular injury in kidney and bladder tissues. Kidney and bladder tissues of rat infected with *S. marcescens* showed increasing level of MDA production. In contrast, phytol treatment significantly (*P* ≤ 0.0005) decreased the MDA production and protected the tissues from lipid peroxidation mediated damages (Figure 8A).

#### Effect of phytol on MPO level

Infiltration of neutrophils in the kidney and bladder tissues of infected rat treated with and without phytol was assessed by estimating the MPO production. MPO is an enzyme produced by neutrophils, which involves in neutralizing the deleterious effect of H_2_O_2_ that cause tissue injury. Assessment levels of MPO in kidney and bladder tissues of infected rats displayed augmented level of MPO production, while, the phytol treatment exhibited a diminution level of MPO production in both kidney and bladder tissues (Figure 8B).

#### Effect of phytol on nitrite content

Reactive nitrogen intermediates are an index of nitrite produced by macrophages and neutrophils with the help of nitric oxide (NO) synthase. Increased level of nitrite production was observed in the *S. marcescens* infected kidney and bladder tissues, whereas, the phytol treatment showed a decreased level of nitrite production in kidney and bladder tissues (Figure 8C).

#### Kidney tissue histology

**Figure 9:**
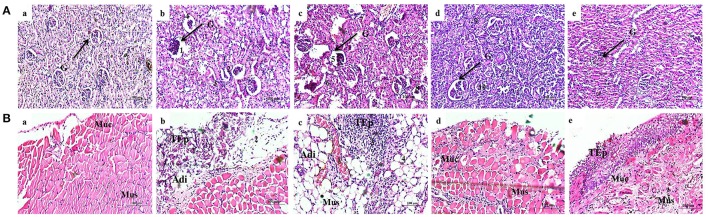
Histopathology analysis of kidney tissue **(A)**. Normal uninfected rat **(a)**, infection control **(b)**, vehicle control **(c)**, phytol treatment (100 mg/kg) **(d)**, phytol treatment (200 mg/kg) **(e)**. 1 and 5, Dilation of Bowman's capsule; 2 and 6, Destruction of renal tubules; 3 and 7, Extensive infiltration of neutrophils; 4 and 8, Shrinkage of glomeruli; 9, Mild infiltration of neutrophils; 10, Well rejuvenated renal tubules; G, Glomeruli. Histopathology analysis of bladder tissue **(B)**. Normal uninfected rat **(a)**, Infection control **(b)**, Vehicle control **(c)**, Phytol treatment (100 mg/kg) **(d)**, Phytol treatment (200 mg/kg) **(e)**. 1 and 3, Extensive infiltration of neutrophils in the bladder wall; 2, Severe abrasion due to infiltration of neutrophils; 4, Larger areolar connective tissue interlaced with the muscular coat; 5, Slight abrasion; Muc, mucosa; Mus, muscularis; TEp, transitional epithelium; Adi, Adipose tissue. Corn oil was used as the vehicle control.

Normal uninfected rat showed normal glomeruli (Figure 9Aa), whereas the rat infected with *S. marcescens* showed severe inflammation and dilation of Bowman's capsule, obliteration of renal tubules and widespread infiltration of neutrophils in the kidney tissue (Figure 9Ab). In vehicle control, infiltration of lymphocytes were observed near glomeruli and is representing an extensive inflammation. Destruction of renal tubules, dilatation of Bowman's capsule and glomeruli shrinkage were also observed (Figure 9Ac). In contrast, the infected rat treated with 100 mg/kg body weight of phytol showed mild infiltration of neutrophils. While, dilated Bowman's space and shrinkage of glomeruli were not observed (Figure 9Ad). Similarly, infected rat treated with 200 mg/kg body weight of phytol showed reduced level of infiltration of neutrophil and is similar to the normal animal control (Figure 9Ae).

#### Bladder tissue histology

Histological section of normal animal bladder showed normal structure of urinary bladder. The three layers of TEp, mucosa and muscularis appear to be normal (Figure 9Ba). In infection control, the increased infiltration of neutrophils was observed in the bladder wall. Also, severe mucosal abrasion was observed due to infiltration of neutrophils (Figure 9Bb). Similarly in vehicle control, marked inflammation and infiltration of neutrophil was observed in the transitional epithelium layer and larger areolar connective tissue was observed (Figure 9Bc). In contrast, the 100 mg/kg body weight of phytol treatment group showed no remarkable inflammation in the transitional epithelium layer, while, slight abrasion was observed in the urothelium (Figure 9Bd). Histological section of urinary bladder in 200 mg/kg body weight of phytol treatment showed clear pathological changes (Figure 9Be).

### Single dose toxicity study

#### Hematological and biochemical parameters

The haematopoietic system is one of the utmost sensitive targets for toxic compounds and considered as a vital index of pathological and physiological status in living systems. Similarly, assessment of biochemical profile acts as valuable indicator to assess the toxic nature of drugs in man and animals. In the pilot single dose toxicity study, no noteworthy difference was observed in the hematological and biochemical profile between the animal control and the phytol treated group (200 mg/kg) (Table 3). Compared to animal control, a slight increament was observed in ALP and SGPT levels in the phytol treated group (200 mg/kg). There was a significant decrease in triglycerides and total cholesterol level in the group treated with 200 mg/kg of phytol, when compared to the animal control.

**Table 3:** Hematological and biochemical profiles of animal control and animal treated with phytol.

**Hematological and biochemical parameters**	**Control**	**Phytol 200 mg/kg**
Hemoglobin (gm/dL)	12.16 ± 1.8	11.84 ± 3.4
WBC (× 10^3^) (μL ^−1^)	7.52 ± 0.6	7.18 ± 1.5
RBC (× 10^6^) (μL^−1^)	3.94 ± 0.6	3.92 ± 1.1
Hematocrit (%)	35.48 ± 7.03	35.58 ± 10.2
Glucose (mg/dL)	80.6 ± 16.1	76.8 ± 15.1
Blood urea (mg/dL)	48.24 ± 4.6	53.2 ± 4.6
Creatinine (mg/dL)	0.542 ± 0.3	0.48 ± 0.07
ALP (IU/L)	107.8 ± 9.8	127.6 ± 23.9
SGOT (U/L)	39.4 ± 7.2	44.4 ± 10.5
SGPT (U/L)	20.2 ± 5.1	27 ± 10.4
Triglycerides (mg/dL)	96.4 ± 6.4	91 ± 17.4
HDL (mg/dL)	16.8 ± 4.1	16.6 ± 1.4
LDL (mg/dL)	113.84 ± 20.3	105.2 ± 10.6
VLDL (mg/dL)	18.96 ± 1.6	18.2 ± 3.4
Total bilirubin (mg/dL)	0.384 ± 0.1	0.48 ± 0.1
Direct bilirubin (mg/dL)	0.246 ± 0.08	0.3 ± 0.06
Indirect bilirubin (mg/dL)	0.124 ± 0.04	0.18 ± 0.07
Total cholesterol (mg/dL)	152.4 ± 21.0	140 ± 8.9
Total Protein (gm/dL)	5.22 ± 0.4	5.14 ± 0.9
Albumin (gm/dL)	3.58 ± 0.4	3.56 ± 0.3
Globulin (gm/dL)	1.64 ± 0.3	1.58 ± 0.5

*Phytol treatment (200 mg/kg) did not show any significance variations in hematological and biochemical profiles, compared to the animal control. Data are expressed as mean ± SD*.

#### Histological evaluation of vital organs of normal and phytol treated animals

**Figure 10:**
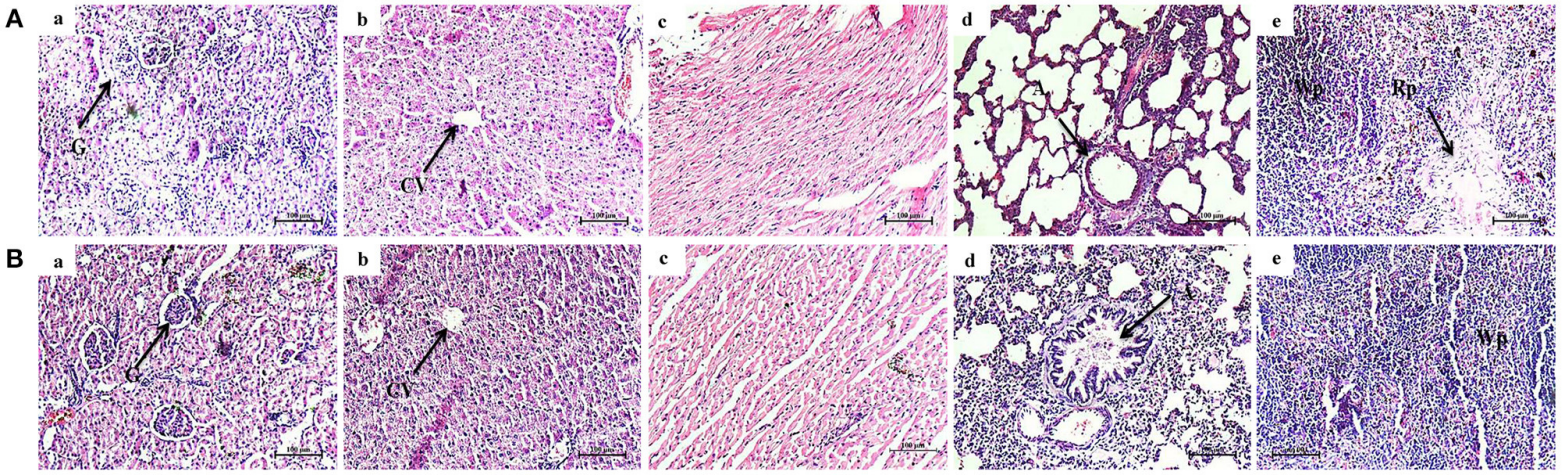
Histopathology analysis of vital organs. Phytol treatment (200 mg/kg) **(B)** did not show any considerable histology varitions in vital organs such as kidney **(a)**, liver **(b)**, heart **(c)**, lungs **(d)**, and spleen **(e)**, compared to the vital organs of animal control **(A)**. G, Glomeruli; CV, Central Vein; A, Alveolar; Wp, White pulp; Rp, Red pulp.

Histological micrographs of kidney from untreated animal showed normal glomeruli size and the proximal and distal convoluted tubules exhibit a normal fine structures (Figure 10Aa), while 200 mg/kg phytol treatment showed intact glomeruli with normal structure (Figure 10Ba). The liver sectioning of control animals portrayed normal architecture and hepatic cells with granulated cytoplasm. The hepatocytes were polygonal shape with a rounded nucleus, arranged in cords with the portal tract exhibiting a normal structure (Figure 10Ab). The rats administered with 200 mg/kg body weight of phytol showed only a moderate degeneration of hepatocytes (Figure 10Bb). Similarly, sections of heart from control and phytol treated animals showed normal muscle fibers with acidophilic cytoplasm and centrally located nuclei (Figures 10A,Bc). The lung sections appears to be normal in phytol treated and control animals with typical alveoli (Figures 10A,Bd). The spleen from control and phytol treated animals showed normal granular hemosiderin pigment predominantly within macrophages in the red pulp. The white pulp containing lymphocytes surrounded by a red pulp (Figures 10A,Be).

## Discussion

UTI is an infection occurred wherever in the urinary system typically exposed to bacterial pathogens. Once bacterial pathogens reach the kidney through ascending infection, they are capable to adhere to the urothelium before raiding the renal tissue with subsequent pyelonephritis (Nickel et al. (1987). Such sort of infections are reported to be caused by Gram-negative bacteria like *P. mirabilis, E. coli, Klebsiella pneumoniae, P. aeruginosa, S. marcescens*, and Gram-positive bacteria such as *Staphylococcus aureus* and *Enterococcus faecalis* (Su et al., 2003; Behzadi et al., 2010; Kaur et al., 2014).

Among which, *S. marcescens* is an important human opportunistic bacterial pathogen, causing numerous nosocomial infections such as respiratory tract infections, blood stream infections, ocular infections and most importantly urinary tract infections (Hejazi and Falkiner, 1997). It secretes array of virulence factors and forms biofilm via signal mediated QS mechanism. In our previous study, we assessed the anti-QS potential of phytol through primary assays such as prodigiosin production, protease inhibition assays and biofilm cells quantification by crystal violet assay (Srinivasan et al., 2016). Nevertheless, the present study further evaluated the potentials of phytol against *S. marcescens* by assessing various virulence assays such as biofilm cells quantification by XTT reduction assay, microscopic analyses of biofilm formation, swarming motility analysis, lipase, hemolysin and EPS quantification assays. In addition, the current study elucidated the molecular mechanism of phytol on QS system in *S. marcescens* through real-time expression analysis and confirmed its *in vivo* protective effect on acute pyelonephritis infection in rat model with satisfactory safety evaluated by single dose toxicity studies.

Biofilms are the aggregation of microorganism, wherein the microbial cells stick to each other on biotic and abiotic surfaces and composed of extracellular DNA, polysaccharides and proteins (Abdel-Aziz and Aeron, 2014). Therefore, we tested the effect of phytol on biofilm formation and EPS production in *S. marcescens* by XTT reduction and EPS quantification assays. The obtained results showed decreasing level of metabolically active cells involved in biofilm formation and EPS production in phytol treatment compared to their respective controls (Figures 2A, 4A). Further, the light and CLSM (2, 2.5, and 3 D) images confirmed the antibiofilm potential of phytol, in which, the 5 and 10 μg/ml of phytol treatment showed disintegration of biofilm formation. Divergently, the control slides showed thick coating of biofilm formation (Figures 3A,B). Our results are going well with the findings of the previous researches, who have reported that the morin reduced the metabolically active cells involved in *Listeria monocytogenes* biofilm formation (Sivaranjani et al., 2016) and marine bacterial extract G-16 effectively inhibited the *S. marcescens* EPS production (Padmavathi et al., 2014).

Several bacterial pathogens simultaneously grow and spread rapidly over a surface through the pattern of movement called swarming motility. This diminishes competition between bacterial cells for nutrients and speeding their growth (Kaiser, 2007). This typical virulent phenomenon in *S. marcescens* plays a vital role in catheter associated urinary tract infections. In this bacterial species the phenomenon of swimming and swarming motility is associated with QS. Hence, an attempt was made to examine the QSI potential of phytol in inhibiting the swarming movement. Results of the current study showed vigorous swarming motility in the untreated *S. marcescens* control plate, wherein the 5 and 10 μg/ml of phytol treatment showed concentration dependent swarming motility inhibition (Figure 3C). Consistent with this result, Srinivasan et al. (2016) have reported that *Piper betle* extract effectively inhibited the *S. marcescens* swarming motility in a concentration dependent manner.

Lipase is the secreted extracellular virulence enzyme in *S. marcescens* and their production is regulated by QS. Hemolysin production is accountable for the pathogenesis of various bacterial pathogens. Hemolysin produced by *S. marcescens* (ShlA), is a group of pore forming toxins, targets the cell membrane permeability (Shimuta et al., 2009). The result of lipase and hemolysin inhibition assays indicated a significant (*P* ≤ 0.0005) decline in lipase and hemolysin production in *S. marcescens* upon treatment with 5 and 10 μg/ml of phytol (Figures 4A,B). Previously, *Anethum graveolens* extract and farnesol were tested for their effects on lipase and hemolysin production in *S. marcescens* and *P. aeruginosa* respectively, and which showed promising lipase and hemolysin inhibitory properties (Hassan Abdel-Rhman et al., 2015; Salini and Pandian, 2015).

Further to understand the anti-QS and antibiofilm potential of phytol at molecular level and to support the outcome of *in vitro* results, the real-time PCR analysis was performed. It is known that *fimA* and *fimC* are the major fimbrial subunits in *S. marcescens*. In 2007, a study done by Labbate et al. disclosed that the *fimA* disruption mutant unable to produce fimbriae and likewise they confirmed the absence of fimbrial structure in *S. marcescens* by electron microscopy. The products of the *flhDC* master operon, FlhD and FlhC are global gene regulators in *S. marcescens*, which expressed several inherent determinants such as cell differentiation, cell division, swimming and swarming motilities (Liu et al., 2000). Therefore, the impact of phytol on the *fimA, fimC, flhC*, and *flhD* gene expression levels were tested and the obtained real-time data showed a substantial downregulation of these fimbrial and motility genes expression in *S. marcescens*. The *bsmB* is a QS controlled virulence gene in *S. marcescens*. Labbate et al. (2007) reported that the *bsmB* mutant lacked biofilm formation, lipase, protease and S-layer protein productions. Phytol treatment decreases the expression level of *bsmB* gene up to 0.36-fold compare to the control. The RssA-RssB (RssA-sensor kinase and RssB -response regulator) is a two component system and it negatively regulates the *S. marcescens* swarming motility. RssB binds directly to the *flhDC* promoter and suppresses the *flhDC* transcription, leading to reduced production of hemolysin and flagellar mediated motilities (Lin et al., 2010). In Ang et al. (2001) stated that the overexpression of *rsmA* gene in *S. marcescens* inhibits the swarming motility and prodigiosin production. The *pigP* is the master transcriptional regulator and which controls the regulation of prodigiosin pigment production in *S. marcescens* under the QS mechanism (Gristwood et al., 2011). RssB binds directly to the promoter region of the pig operon, leading to negative regulation of prodigiosin production (Soo et al., 2014). The outcome of real-time data showed upregulation of *rssB* and *rsmA* genes expression and support the *in vitro* data of hemolysin, swarming motility and prodigiosin inhibition due to their binding on *flhDC* and *pigP* promoter regions. Likewise, phytol decreases the expression level of *pigP* gene upto 0.48 fold compare to the control. ShlA is a key virulence factor of *S. marcescens*, which has shown to wield cytotoxic effects on fibroblasts and epithelial cells (Di Venanzio et al., 2014) and *shlBA* mutant strains were extremely reduced in virulence in mice, *Drosophila melanogaster* and *Caenorhabditis elegans* models (Kurz et al., 2003). In *S. marcescens*, hemolysis and swarming motility are co-regulated (Shanks et al., 2013). In the current study the phytol inhibited the hemolysin production along with swarming motility inhibition. Similarly, the real-time data showed downregulation of *shlA* gene upon treatment with phytol (Figure 5).

The recent reports stated that the QS mediated virulence factors are very important for establishment of successful UTI infection in animal models (Kumar et al., 2009; Gupta et al., 2013b, 2016; Saini et al., 2015). Only limited studies specified the pathogenesis of *S. marcescens* in animal models and also no reports are available on the protective effect of plant extracts or pure compounds against *S. marcescens* associated infection in animal models. To the best of our knowledge, the present study is the first of its kind has been made with a prime objective to establish the *S. marcescens* associated acute pyelonephritis in rat and assessing the protective effect of phytol against acute pyelonephritis induced rat.

After successful establishment of acute pyelonephritis in rat model, the bacterial count in phytol treated and untreated rats were quantified by bacteriological assay. The infected control had 8.28 × 10^4^, 7.2 × 10^4^, and 3.72 × 10^4^ CFU in kidney, bladder and urine samples, respectively compare to the 200 mg/kg body weight of phytol treated group in which 1.78 × 10^4^, 0.96 × 10^4^, and 0.5 × 10^4^ CFU were observed in kidney, bladder and urine samples, respectively (Figures 6B–D). This corresponds to nearly 4.6, 7.5 and 7.4 fold decrease in bacterial count in phytol (200 mg/kg body weight) treated kidney, bladder and urine samples respectively, compared to the infected control. These results correlate with the findings of Hvidberg et al. (2000), who have reported that the antibiotic gentamicin treatment significantly decreased the bacterial count in kidney, bladder and urine samples in UTI induced mice compare to the infection control.

Colonization of bacterial pathogens on host tissue during the early stage of infection is an essential factor for the establishment of very infection. Virulence factors produced by the bacterial pathogens help in the host colonization and subsequent infection progress. The extracellular virulence enzyme protease plays a pivotal role in the pathogenesis of *S. marcescens* during infection and induces interleukin-6 and interleukin-8 mRNA expression through protease-activated receptor 2 (PAR-2) (Kida et al., 2007). A study made by Lyerly and Kreger (1983) state that the highly purified protease enzyme obtained from *S. marcescens* induced the acute pneumonia in mice and guinea pigs. A finding made by Ishii et al. (2014) revealed that the protease intricate in the pathogenesis of *S. marcescens* and leads to a huge loss of hemolymph in silkworm larvae. Like protease, the extracellular lipase enzyme also an extensive virulence factor and which involved in the pathogenesis of *S. marcescens* (Hejazi and Falkiner, 1997). Both of these virulence enzyme productions are controlled by the QS mechanism (Labbate et al., 2007). In support, the result stated by Elsheikh et al. (1987) indicated that the virulence enzyme protease enhances the pathogenesis of *P. aeruginosa* in experimental mouse burn infection. In Gupta et al. (2013a) suggested that the QS mediated virulence enzymes such as protease and elastase are involved in the establishment and colonization of *P. aeruginosa* in mice during experimental UTI. Therefore, the inhibitory effect of phytol on virulence enzyme production in rat acute pyelonephritis model was evaluated. As expected the phytol treatment showed decreased level of protease and lipase enzymes production in both kidney and bladder tissues compared to the infected and vehicle controls (Figure 7). The extreme reduction in virulence enzyme productions of kidney and bladder tissues in phytol treated groups is go well with bacteriological assay. Hence, it is envisaged that the decreasing level of virulence enzymes in phytol treated groups might be due to the decreasing level of invading *S. marcescens* cells.

MDA is an indicator of lipid peroxidation and which is a steady product of oxidative stress of reactive oxygen species on unsaturated fatty acid, a vital constituent of cell membrane. In the current study, the kidney and bladder tissues from infected and vehicle control groups showed a substantial increase in MDA level on 5th p.i.d, whereas the phytol treated groups showed decreasing level of MDA production in kidney and bladder tissues (Figure 8A). Consistent with our results, synergistic combination of azithromycin and ciprofloxacin has been shown to decrease the MDA level in kidney tissue homogenates of *P. aeruginosa* infected mice on the 3rd and 5th p.i.d (Saini et al., 2015).

MPO is an enzyme deposited in azurophilic granules of polymorphonuclear neutrophils and macrophages, which released during inflammatory process and oxidative stress into extracellular fluid. The MPO is a possible pathological marker for the confirmation of inflammation (Loria et al., 2008). In the present study, the MPO level was considerably low in case of infected rats treated with phytol compare to the infected and vehicle controls in both kidney and bladder tissues (Figure 8B). The results of MPO assay go well with the findings of Vadekeetil et al. (2016), who have reported that the ajoene-ciprofloxacin combination effectively decreasing the MPO production in the mice infected from *P. aeruginosa* biofilm associated murine acute pyelonephritis.

NO is produced by a different cell types by NO synthases, which are involved in the inflammatory processes. Stimulation of NO production during inflammatory progression signifies a protection mechanism against invading bacterial pathogens, however extreme formation of NO has also been involved in host tissue injury (Van Der Vliet et al., 1997). A significant (*P* ≤ 0.0005) decline of nitrite in the levels of protein was observed in kidney and bladder tissues of phytol treatment groups compare to the infection and vehicle controls. Similar to the observed results, recently the combination therapy with ajoene and ciprofloxacin has been found to show decreasing level of NO production in mice infected with *P. aeruginosa* (Vadekeetil et al., 2016).

To support the decreasing level of virulence enzymes and inflammatory markers in phytol treated groups, the histopathology analysis was done. Kidney sections of the normal uninfected rats looked histologically normal with no substantial pathological variations (Figure 9Aa). The kidney sections of infection and vehicle control rats had extensive infiltration of neutrophils with destruction of renal tubules and shrinkage of glomeruli (Figures 9Ab,c). In case of 100 mg/kg body weight of phytol treated group, a mild infiltration of neutrophils was noted and 200 mg/kg body weight of phytol treatment showed no considerable pathological changes (Figures 9Ad,e). Recently, Balamurugan et al. (2015) found that the treatment of UTI^QQ^ with gentamicin against rats infected with *S. aureus* showed minimal dilatation of renal tubules with no considerable pathological changes in kidney section. The bladder histology section of infection and vehicle controls showed extensive infiltration of neutrophils with severe abrasion in transitional epithelium (Figures 9Bb,c). In contrast, the uninfected rat and infected rat treated with phytol showed no considerable pathological changes (Figures 9Ba,d,e). Outcome of this bladder histology supports the results of Sabharwal et al. (2016), who have not observed any adverse pathological changes in divalent flagellin treated mice bladder tissue.

The toxicological property of phytol has been tested in different animal models for different clinical applications (Hidiroglou and Jenkins, 1972; McGinty et al., 2010). The acute oral LD_50_ of phytol in rats was described to be more than 5.0 g/kg body weight (McGinty et al., 2010). However, the rats were dosed for 28-day in sub chronic toxicity study showed the no-observed-adverse-effect-level (NOAEL) of phytol to be 500 mg/kg/day, based on organ weight changes. In contrast, the rats were dosed for a longer period of time (52–108 days) in a one-generation reproductive toxicity study, the lowest-observed-adverse-effect level (LOAEL) of phytol was to be 250 mg/kg/day, based on renal changes in male and female rats (Api et al., 2016). The overall mammalian toxicity of phytol is considered to be low only in least concentration. Hence, the protective effect of phytol was tested against *S. marcescens* associated acute pyelonephritis infection at the concentration of 100 and 200 mg/kg. On the other hand, we assessed the toxic effect of phytol (200 mg/kg) by single dose acute toxicity study. No significant differences were observed in the hematological profile of phytol treated group compared to the animal control (Table 3). The oral administration of phytol in rats did not show any significant changes in biochemical profile when compared to the animal control group (Table 3). However, an increase in ALP and SGPT serum blood levels were observed in the phytol treatment. ALP and SGPT are generally used as markers for liver function and indicators of liver toxicity. ALP and SGPT levels elevate in the blood when the hepatic cellular permeability is changed or cellular injury occurs in liver. The histopathological analysis of vital organs (Kidney, Liver, Heart, Lungs, and Spleen) in phytol treated group did not show any adverse pathological effects compared to the animal control, except liver section (Figure 10). The liver section of phytol treatment showed moderate degeneration of hepatocytes (Figure 10Bb) and it was due to the increasing level of ALP and SGPT. The degeneration of hepatocytes and increasing level of liver enzymes support the outcome of Mackie et al. (2009), who have reported that the phytol induced the hepatotoxicity in mice.

To the best of our knowledge, this is the pioneering study annex the anti-QS and antibiofilm capability of phytol in the counteractive action on *S. marcescens* infection through the serious of virulence inhibition assays. The real-time analysis disclosed the molecular mechanism of phytol on QS intervened virulence factors productions in *S. marcescens*. Further, the *S. marcescens* associated acute pyelonephritis infection in rat model unveiled the protective effect of phytol by reducing the bacterial counts, virulence enzymes and inflammatory markers productions with adequate safety. Therefore, the utilization of phytol is promising in the advancement of novel antipathogenic medications to control acute pyelonephritis infection caused by *S. marcescens*. However, further studies will be needed to reveal the mode of action of phytol against *S. marcescens* associated acute pyelonephritis infection.

## Author contributions

AV and RS conceived and designed the research; RS, AK, and VK performed the experiments; RM, KR, and GA offered advice and technical assistance for carrying out the studies on experimental animals; AV and RS analyzed the data; AV, SK, and KR contributed reagents/materials/analysis tools; RS wrote the paper and AV approved the manuscript after careful analysis.

### Conflict of interest statement

The authors declare that the research was conducted in the absence of any commercial or financial relationships that could be construed as a potential conflict of interest.

## Supplementary material

Supplementary Figure 1 PCR amplification for the checking the primer efficiencies of QS controlled virulence genes in *S. marcescens*.
