# Supplementary figures and images for: Exploring the Anti-quorum Sensing and Antibiofilm Efficacy of Phytol against Serratia marcescens Associated Acute Pyelonephritis Infection in Wistar Rats
Source: Front Cell Infect Microbiol. 2017 Dec 5;7:498. doi: 10.3389/fcimb.2017.00498 (PMC5723315; doi:10.3389/fcimb.2017.00498)

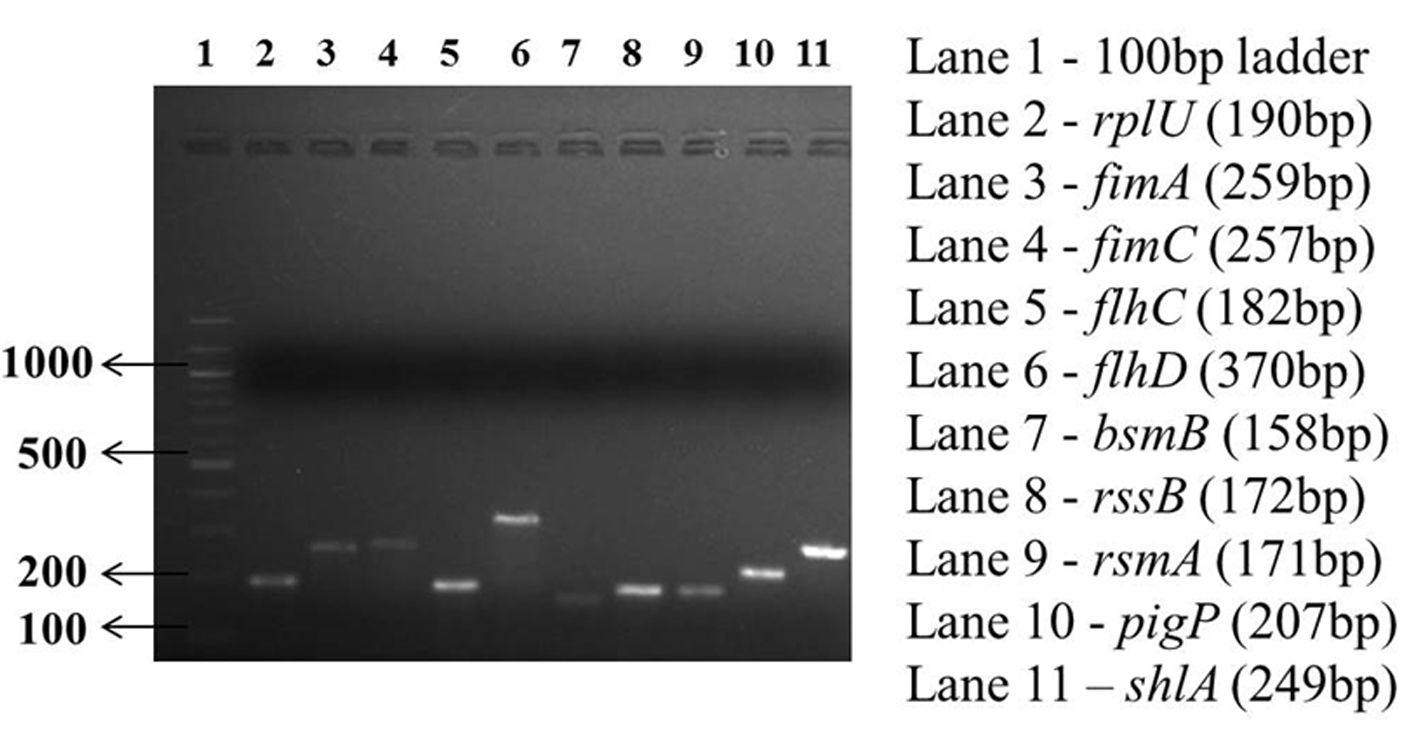

Supplement: Supplementary Figure 1 — PCR amplification for the checking the primer efficiencies of QS controlled virulence genes in S. marcescens. [file Image1.JPEG]
